# A Capacitive Pressure Sensor Interface IC with Wireless Power and Data Transfer

**DOI:** 10.3390/mi11100897

**Published:** 2020-09-27

**Authors:** Chaoping Zhang, Robert Gallichan, David M. Budgett, Daniel McCormick

**Affiliations:** Auckland Bioengineering Institute, The University of Auckland, Auckland 1010, New Zealand; r.gallichan@auckland.ac.nz (R.G.); d.budgett@auckland.ac.nz (D.M.B.); d.mccormick@auckland.ac.nz (D.M.)

**Keywords:** AFE, ADC, BGR, LDO, oscillator, rectifier, SC sampling, SC op-amp

## Abstract

This paper presents a capacitive pressure sensor interface circuit design in 180 nm XH018 CMOS technology for an implantable capacitive pressure sensor, which has a wireless power supply and wireless data transfer function. It integrates full-bridge rectifiers, shorting control switches, low-dropout regulators, bandgap references, analog front end, single slope analog to digital converter (ADC), I2C, and an RC oscillator. The low-dropout regulators regulate the wireless power supply coming from the rectifier and provide a stable and accurate 1.8 V DC voltage to other blocks. The capacitance of the pressure sensor is sampled to a discrete voltage by the analog front end. The single slope ADC converts the discrete voltage into 11 bits of digital data, which is then converted into 1 kbps serial data out by the I2C block. The “1” of serial data is modulated to a 500 kHz digital signal that is used to control the shorting switch for wireless data transfer via inductive back scatter. This capacitive pressure sensor interface IC has a resolution of 0.98 mmHg (1.4 fF), average total power consumption of 7.8 mW, and ±3.2% accuracy at the worst case under a −20 to 80 °C temperature range, which improves to ±0.86% when operated between 20 and 60 °C.

## 1. Introduction

Pressure is an essential indicator of patient health and disease progression as it is highly regulated in different organs in the human body such as the bladder, heart, eye, and brain [1]. Capacitive pressure sensors are widely used to detect these pressure changes as they are often cited with the advantage of higher-pressure sensitivity [2], lower power consumption, and lower temperature drift [1,3] than piezoresistive sensing, making them a good choice for implantable medical applications. For implantable pressure monitoring, ±1 mmHg resolution at the clinically normal pressure range is commonly accepted [1].

For a fully implantable device, sensor, sensor interface circuitry, wireless data telemetry, and wireless power are typically needed. To interface with a capacitive pressure sensor, the combination of analog front end (AFE) (commonly capacitance to voltage converter) and analog to digital converter (ADC) topology is typically utilized. For the AFE, there are a variety of choices, such as capacitance to frequency [4,5,6], capacitance to time [7,8,9], and switched-capacitor (SC) sampling [10,11,12,13]. For the ADC that is commonly used in this application, there are dual-slope [14,15,16,17,18], successive-approximation (SAR) [12,19,20,21] and delta-sigma [22,23,24,25,26] ADCs. There are also some interface methods such as oscillation frequency [27] that do not need an ADC. This method offers a low complexity at the disadvantages of poor sensitivity, frequency instability, and poor stray capacitance immunity [28].

Considering wireless powering and data links, the vast majority of devices utilize RF or inductive methods such as in [7,13]. Recently, ultrasonic links have been proposed for both power recovery and data transmission due to the advantage of lower tissue attenuation and highly directive focusing [12].

In this paper, SC AFE plus single-slope ADC with inductive power supply and data transfer are investigated at the system level, described in detail at the circuit and transistor level, and also presented with some experimental results. It achieves full functionality on a monolithic chip with features of capacitive pressure sensing, wireless power supply, and wireless data transfer. It has a trade-off between resolution and power consumption.

The following sections present the complete design of an integrated capacitance interface. The interface’s principle and a detailed description of each circuit block are introduced in Section 2. Post layout simulation results are presented in Section 3 with conclusions drawn in Section 4.

## 2. System Overview and Circuit Implementation

**Figure 1:**
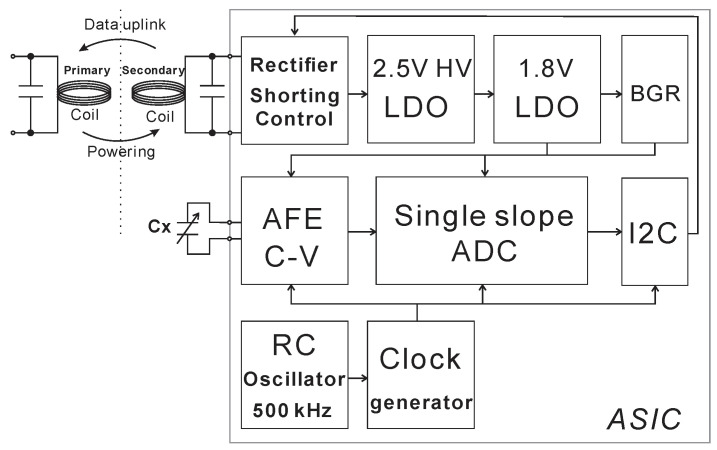
The system architecture of the capacitive pressure sensor interface showing the sense (Cx) capacitor.

**Figure 2:**
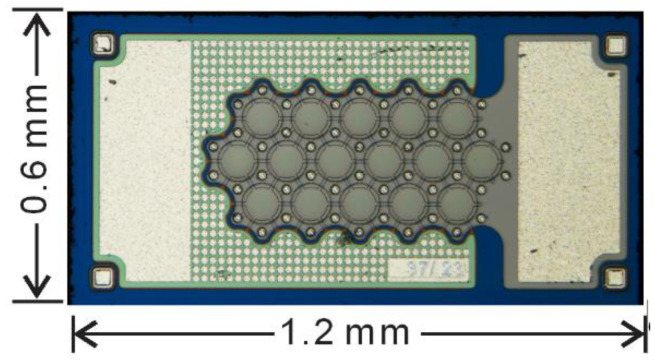
Capacitive pressure sensor die from Protron with which the interface is designed to work.

The system block diagram of this design is shown in Figure 1. It interfaces with an external capacitive MEMS pressure sensor (Cx) available from Protron Mikrotechnik [29] and shown in Figure 2. This pressure sensor can measure approximately 300–1000 mmHg ambient pressure corresponding to a 6–7 pF capacitance range according to the datasheet from Protron Mikrotechnik. The external (secondary) coil provides power to the ASIC and also works as a near-field antenna for transmitting out the data. All bias voltages and bias currents are provided by the bandgap reference (BGR) block. The switching clocks are generated by the internal RC oscillator.

### 2.1. The Rectifier and Shorting Control Circuit

**Figure 3:**
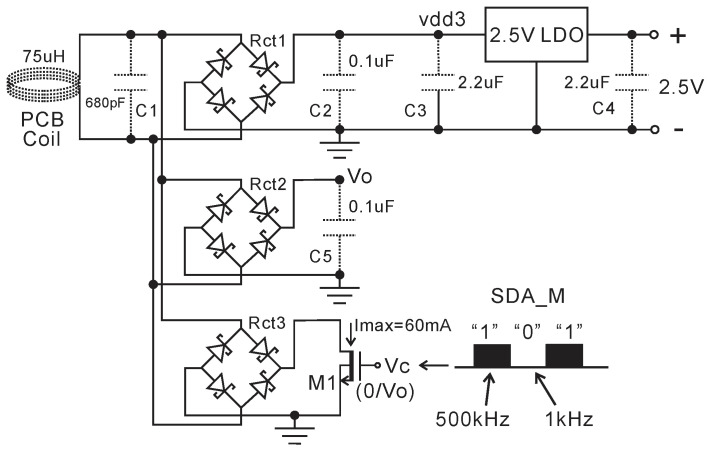
The rectifiers and shorting control circuit. The components shown with a dotted line are external.

**Figure 4:**
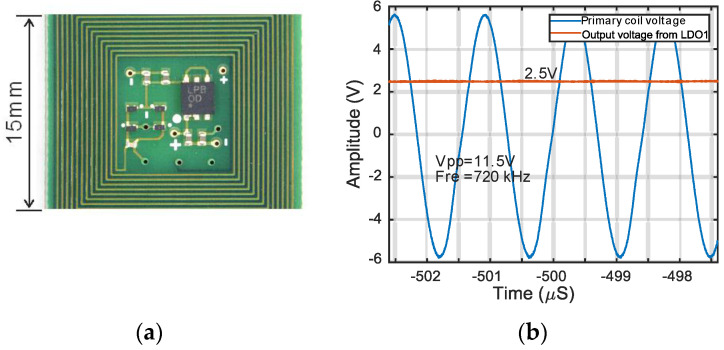
(**a**) A six-layer PCB (printed circuit board) for inductive power transfer testing with a full bridge rectifier and commercial low-dropout regulator (ADP7118AUJZ-2.5-R7). (**b**) Measured regulated 2.5 V DC output voltage from the PCB coil (15 × 15 mm^2^) with a 720 kHz transmit frequency.

Figure 3 shows the inductive power transfer (IPT) and shorting control circuit. The 2.5 V high voltage (HV) low-dropout (LDO) regulator regulates the output of the four Schottky diodes rectifier to 2.5 V DC output and also protects other blocks from high voltage. An LTspice simulation and experiment have been done with discrete components in order to choose the proper value of inductance (PCB coil) and capacitance (capacitor C1) to make them resonate and receive maximum power at 720 kHz. The experimental results in Figure 4b show that the desired resonant working point can be obtained by choosing approximately 720 kHz transmit frequency when using a 72 µH coil and 680 pF capacitor C1, which is consistent with our previous theoretical analysis from $f=1/(2\pi \sqrt{LC})\;$. The full-bridge rectifier and HV LDO now have been integrated into the current ASIC, as shown in Figure 3. The 2.5 V HV LDO will be introduced later.

In Figure 3, Rct1 is responsible for providing power to the other blocks. The extra rectifier, Rct2, can provide a higher control logical high voltage to the gate of the shorting switch M1 (18 V HV transistor, Vthn = 1.6 V, Vthp = 1.9 V) compared to using the control high voltage from the output of Rct1 and thus guarantee M1 to operate in the triode region. The control logical signal Vc comes from the modulated serial data (SDA) of the I2C output, SDA_M. The current draw at “1” is approximately 60 mA, which can be detected at the primary coil side shown in Figure 1.

### 2.2. 2.5V HV LDO Circuit

**Figure 5:**
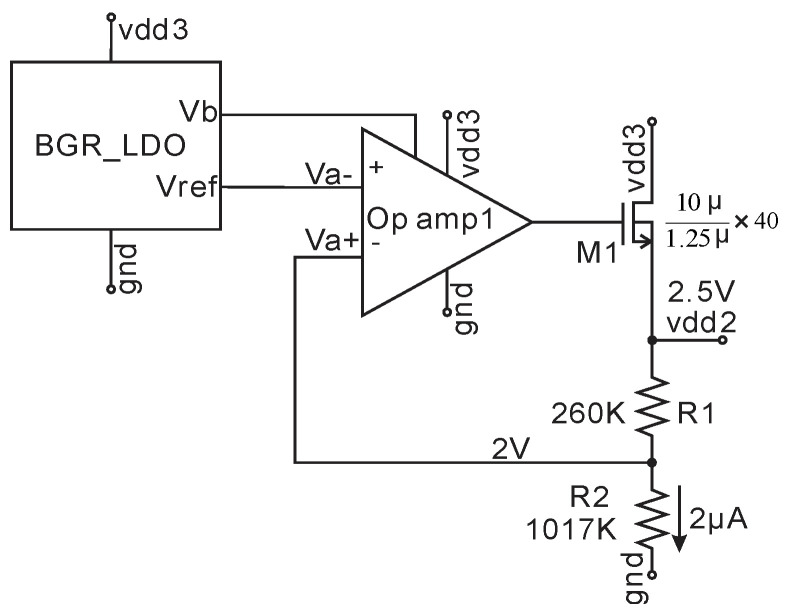
Schematic of the 2.5V high voltage (HV) low dropout (LDO).

**Figure 6:**
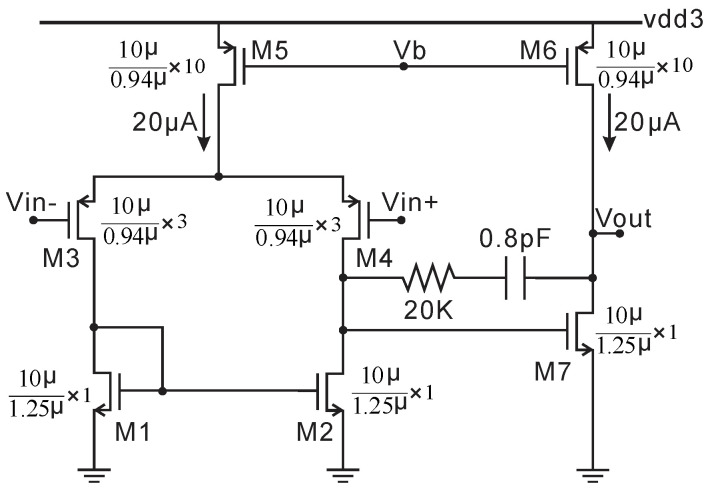
Schematic of op-amp1 used in the 2.5 V HV LDO.

The 2.5 V HV LDO circuit is shown in Figure 5. The input, vdd3, is from the output of Rect1, and the output of this HV LDO is vdd2 (2.5 V). The bandgap reference block provides bias voltage Vb and reference voltage Vref for op-amp1 depicted in Figure 6. All transistors in HV LDO are 18 V HV transistors. Under no-load Va+ is equal to Va- and the LDO output is

(1) $$vdd2=\frac{R1+R2}{R2}Vref=2.5V.$$

The feedback loop holds vdd2 constant, which provides a regulated stable supply with a maximum 4 mA current.

**Figure 7:**
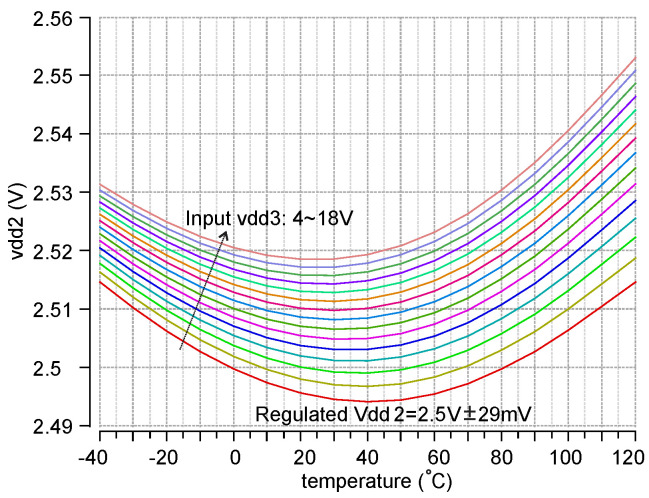
Post layout transient analysis sweep of the 2.5 HV LDO with 2 KHz and 1 mA switching load.

A 4–18 V and 2 kHz sine wave input signal emulating the extreme voltage output from rect1 is used to test the HV LDO over the temperature range −40 to 120 °C. The load is a 2.5 KΩ resistor in series with a 2 kHz switching (emulating 1 mA digital circuits load) from vdd2 to ground, which is not drawn in Figure 5. The simulation result is shown in Figure 7. The regulated voltage vdd2 is 2.5V ± 29 mV.

**Figure 8:**
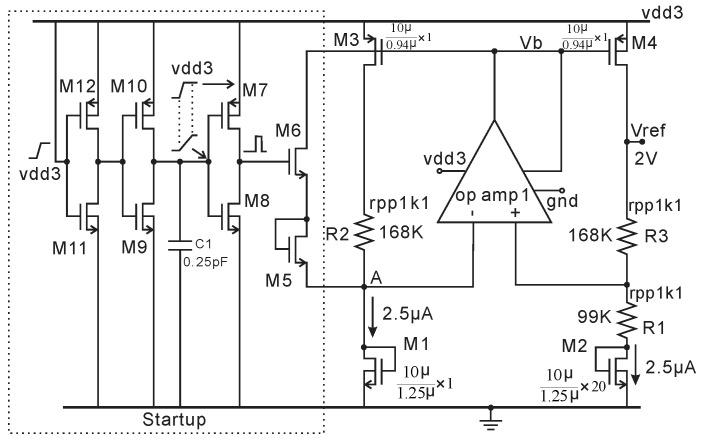
Schematic of bandgap reference (BGR) for HV LDO. R2 is added to reduce the current mismatch through M3 and M4.

The bandgap schematic used in this HV LDO is illustrated in Figure 8, which is modified from [30]. In this circuit, the BGR voltage is about 2 V and provided by the HV NMOS transistors M1 and M2 rather than BJT (bipolar junction transistor). The transistors M5–M12 constitute of a new zero-power start-up circuit for HV BGR. First, we assume the BGR does not start, i.e., when power is on, M3 and M4 are off, and their gate voltage is vdd3. At the same time, the power-on signal is delayed by two inverters (M9–M12) and one capacitor (C1), which is transferred into a pulse signal by the inverters M7 and M8. M6 and the diode-connected transistor M5 will be turned on by the pulse, which will inject current into M1. This will force the op-amp differential input to be negative and the output to go low, ensuring a correct start-up of the BGR, and the startup circuit (specifically, M6) will be completely turned off after the start-up pulse. The reference voltage is

(2) $$V_{ref}=V_{GS1}+\frac{R_{3}}{R_{1}}\Delta V_{GS1,2}=2V,$$

where the ratio of R3/R1 is temperature independent due to the same material (P+ ploy). The first linear temperature-dependent term from the Taylor series of V_GS1_ with temperature can be cancelled by (R3/R1) × ∆VGS1,2. The two-stage op-amp schematic employed in this BGR is the same as the op-amp1 shown in Figure 6. The post layout transient analysis of Vref over a temperature range of −40 to 125 °C with supply voltage steps from 4 to 18 V is 2 V ± 24 mV.

The core of capacitance to the digital converter is designed with 1.8 V transistors and requires an accurate reference voltage and an accurate 1.8 V supply. Therefore, a second 1.8 V LDO designed with 3.3 V transistors and with the power supplied from the 2.5 V HV LDO output (vdd2) is used to power the core. The topology of this 1.8 V LDO is the same as that of the 2.5 V HV LDO. We designed the 1.8 V LDO in a previous chip [31] and directly integrated it in the current ASIC.

**Figure 9:**
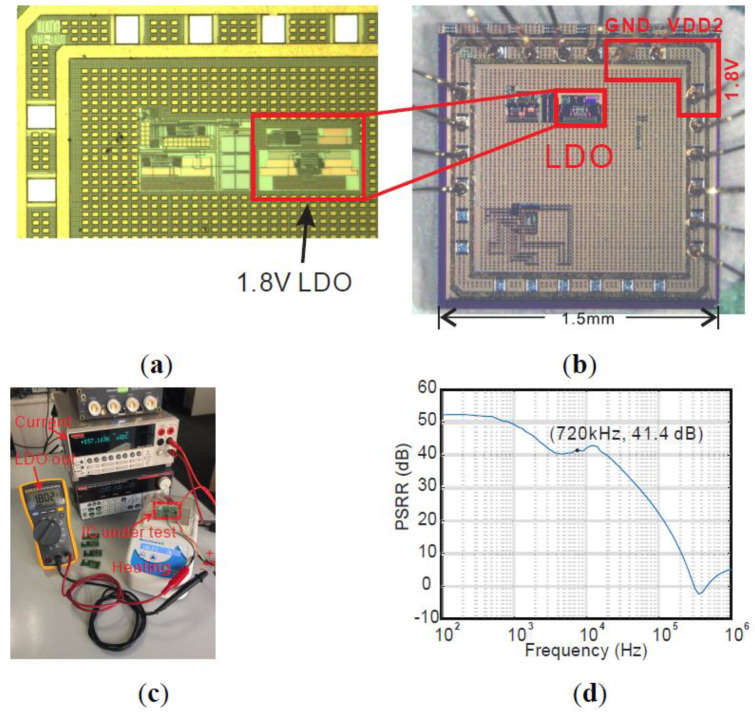
(**a**) Micrograph of the previously fabricated 1.8 V LDO in 180 nm XH018 technology. (**b**) Directly wire-bonded die on a gold pad PCB. (**c**) 1.8 V LDO test setup. (**d**) Measured power supply rejection ratio (PSRR) of 1.8 V LDO with a 0.1 µF capacitor load.

The testing results of the previous 1.8 V LDO are described below. We used the Wire Bonder HB10 to wire bond the IC die Figure 9a directly onto the PCB with a 17 µm gold wire, as shown in Figure 9b. Then, the 1.8 V LDO was tested over a range of DC supply voltages and temperatures. The setup is shown in Figure 9c. The measured 1.8 V LDO output was 1.8 V ± 2 mV when the power supply changed from 1.9 to 3.6 V and the temperature changed from 27 to 100 °C. The measured average current consumption of this LDO from three IC samples is 48 µA (5.5 µA greater than simulation shown in [31]) with room temperature and increased to 57 µA when the temperature rises to 100 °C. The power supply rejection ratio (PSRR) of 1.8 V LDO was tested by applying 1.9–3.6 V sine wave voltage to the supply with a frequency sweep using a signal generator and frequency response analysis from the Clevercope CS448 and a power amplifier CS1070. The measured PSRR is depicted in Figure 9d. The PSRR at the wireless power frequency of 720 kHz with a 0.1 µF capacitor load is 41.4 dB.

### 2.3. Analog Front End (AFE)

**Figure 10:**
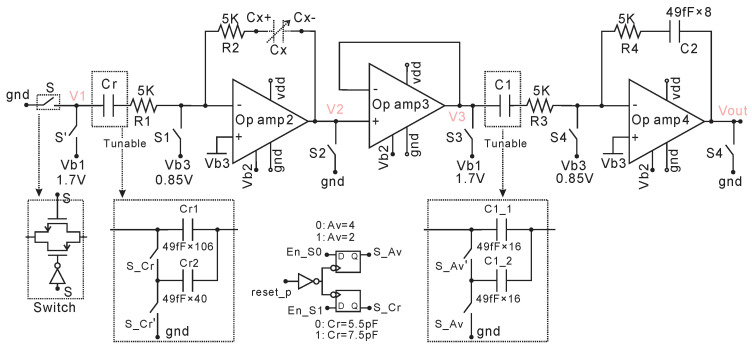
The analog front end (AFE).

**Figure 11:**
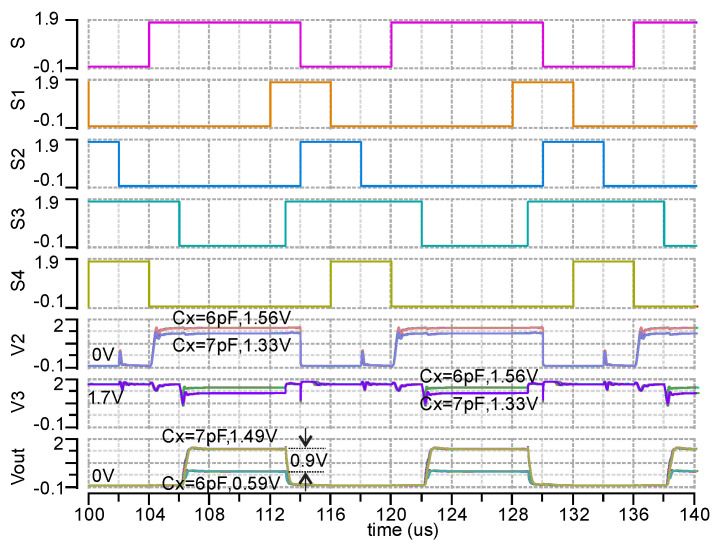
Post layout simulation of AFE over a −40 to 120 °C temperature range with 1.8 V supply, Cref = 5.5 pF and Av = 4. Results show that the Vout changes approximately 900 mV for a 6 to 7 pF capacitance range. Temperature dependence is low due to the use of the BGR bias source and SC sampling method.

The analog front end (AFE), i.e., SC capacitance to voltage converter, is shown in Figure 10. It consists of a switched capacitor sampling circuit (op amp2), buffer (op amp3), and switched capacitor op-amp (op amp4). The op amp2 and op amp4 have the same structure as op amp1 shown in Figure 6. Op amp3 is an NMOS input two-stage op-amp. All switches are made by the transmission gate shown at the bottom left in Figure 10. The control signal of switches S1–S4 is shown in Figure 11, which is generated with an RC oscillator and several digital circuits. Some delays in the control signal and resistors R1–R4 are used in order to reduce current leakage and switching noise. The switching noise at the input of op amp 2 and op amp 4 can reach up to 2.5 V without using this method, which exceeds the maximum transistor operating voltage 1.98 V and thus can reduce transistors’ lifetime or cause direct damage. It should be noted that the sampling frequency, 62.5 kHz (16us), is chosen in order to reduce the current leakage from the Cx+ pad while switch S1 is open (S1 = 0). This total current leakage (or voltage drop) is proportional to time. Thus, the higher the sampling frequency, the lower the total current leakage (voltage drop) will be at the pad Cx+. With the 62.5 kHz sampling frequency, the voltage drop simulated with pads is 57 uV at point Cx+ and 570 uV at Vout. With lower sampling frequency such as 1 kHz, the voltage drop at Vout can go hundreds of millivolts, which would affect the accuracy of the AFE dramatically. This current leakage phenomenon is caused by the discharging of Cx and Cr through the ESD pads, which can only be found when the AFE is simulating with ESD pads. In addition, the reference capacitance (Cr) and amplification gain capacitance (C1) are tunable by manually connecting En_S0 and EN_S1 pins to gnd or vdd, as shown at the bottom in Figure 10. For the specific sensor (Cx: 6 pF~7 pF) in this paper, we can choose Cr = 5.5 pF and Av = 4 to get the best resolution by simulating the EN_S0 = gnd, EN_S1 = gnd. In general, this AFE has two functions. One is capacitance to voltage sampling accomplished from V1 to V2. The sampling voltage V2 can be expressed as,

(3) $$V_{2}=V_{b1}\frac{C_{r}}{C_{x}}=1.7V\cdot \frac{5.5pF}{C_{x}}.$$

When the sensor Cx changes from 6 to 7 pF, V2 decreases from 1.558 to 1.336 V in theory. The post layout simulated result of V2 is 1.556 V ± 5.9 mV to 1.335 V ± 5.0 mV when Cx changes from 6 to 7 pF and the temperature changes from −40 to 120 °C, as shown in Figure 11-V2. The buffer, op amp3, is used to shift the DC voltage from 0 V in V2 to 1.7 V so that the sampling voltage can be amplified within 1.8 V. The second function of the AFE is voltage amplification from V3 to Vout,

(4) $$\begin{array}{cc} V_{out} & =A_{V}(V_{b1}-V_{2})\\ & =\frac{C_{1}}{C_{2}}(V_{b1}-V_{b1}\frac{C_{r}}{C_{x}})=4\cdot 1.7V\cdot (1-\frac{5.5pF}{C_{x}}) \end{array}$$

With the condition $0.2V\leq V_{out}\leq 1.8V$, we can derive the measurable Cx range: $5.67pF\leq C_{x}\leq 7.48pF$. When the sensor Cx changes from 6 to 7 pF, the Vout increases from 0.567 to 1.457 V in theory. The simulated Vout is 0.589 V ± 10.5 mV to 1.495 V ± 13 mV when Cx changes from 6 to 7 pF with the temperature varying from −40 to 120 °C, as shown in Figure 11, Vout. Thus, the sensitivity of the AFE is 0.9 mV/fF. In addition, if connecting EN_S0 = vdd (Av = 2), EN_S1 = gnd (Cr = 5.5 pF), the measurable Cx range is wider: 5.8–11.6 pF but with smaller sensitivity. With EN_S0 = gnd (Av = 4), EN_S1 = vdd (Cr = 7.5 pF), the measurable Cx range is 7.7–10.2 pF. Connecting EN_S0 = vdd (Av = 2), EN_S1 = vdd (Cr = 7.5 pF), the measurable Cx range is 8–15.9 pF. Thus, this C-D converter has a measurable range of 5.7–15.9 pF.

### 2.4. Single Slope ADC

**Figure 12:**
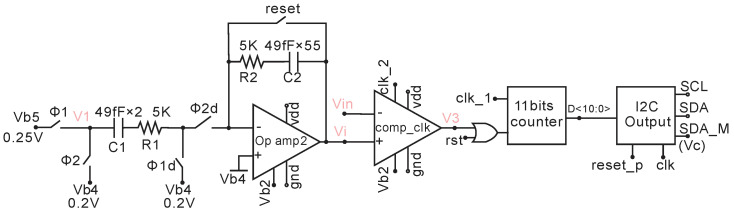
Single slope ADC with I2C output. The analog to digital converter (ADC) uses a switched capacitor integrator and clocked comparator. The integrator voltage per step (clock Φ2) is 1.3 mV.

The AFE output voltage is digitized by a single slope ADC, as shown in Figure 12. This ADC utilizes a switched capacitor integrator, which is independent of temperature variation and thus has the same temperature-independent property as a dual-slope ADC. The control signals for this integrator are Φ1, Φ1d, Φ2, and Φ2d. They are non-overlapping clock signals generated from a clock generator circuit [32], which is not shown here. The integrating voltage per step from V1 to Vi can be expressed as,

(5) $$V_{i}=(V_{b5}-V_{b4})\frac{C_{1}}{C_{2}}=50mV\cdot \frac{2}{55}=1.82mV.$$

**Figure 13:**
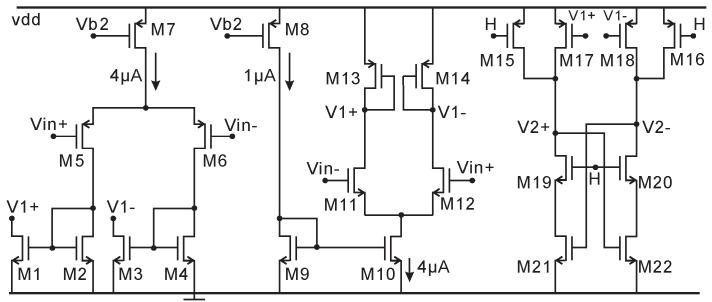
Rail-to-rail input clocked comparator used in the single-slope ADC.

However, the post layout simulated result for Vi is 1.3 mV due to the coupling parasitic capacitance of C2, which is estimated to be 1.07 pF. The schematic of the clocked comparator is shown in Figure 13.

**Figure 14:**
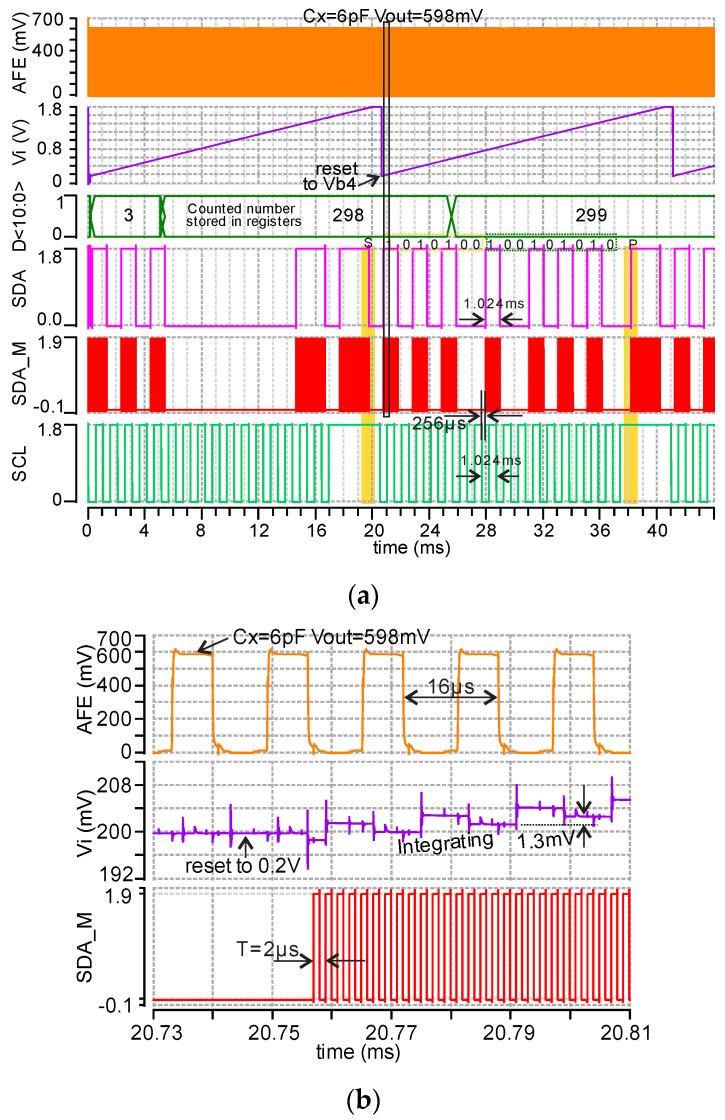
(**a**) Post layout simulation of ADC together with AFE and I2C when the capacitive MEMS pressure sensor (Cx) = 6 pF. The C-V output voltage is 0.59 V and the ADC digital decimal output is 299. (**b**) Zoom of AFE output, Vi (Integrator output), and SDA_M from 20.73 to 20.81 ms in (**a**). The simulated rms total current consumption is 180 uA.

The rail-to-rail input structure is used for this comparator because the input voltage (Vi) varies from 0.2 to 1.8 V. There is also an SR latch connecting with V2+ and V2-, which is not shown in Figure 13 for simplicity. The post layout transient simulation results of ADC with AFE and I2C at 6 pF input capacitance are presented in Figure 14. The AFE is continuously working with 598 mV output when Cx = 6 pF, as shown in Figure 14a-(AFE), and its zoomed view in Figure 14b-(AFE). The voltage Vi and counter are initially reset to Vb4 (0.2 V, as shown in Figure 14b-(Vi) and 0 respectively by a reset signal at the beginning of every byte. Then, Vi is integrating from 0.2 V to its maximum voltage near to vdd, and at the same time, the counter starts to count the clk1 until Vi is higher than Vin (AFE output). Then, the counted number (D<10:0>) is stored in an 11-bit temporary register (shown in Figure 14a-D<10:0>) and transferred to a serial output (SDA) by the I2C digital circuit. A shorting control signal SDA_M (Vc) is also generated to control the shorting rectifier in Figure 3. Cycle by cycle details for the AFE, Vi, and SDA_M can be seen in more detail in Figure 14b.

### 2.5. BGR for Voltage Bias

**Figure 15:**
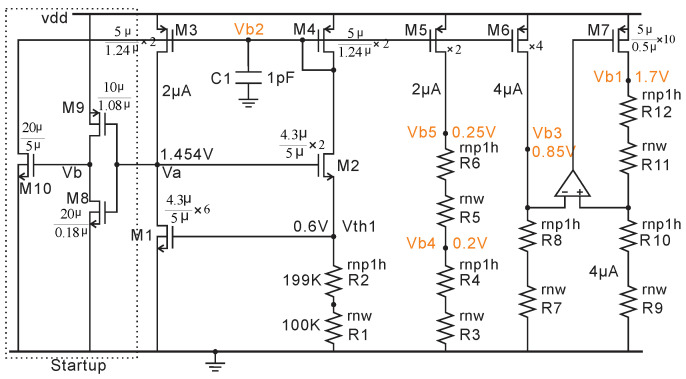
BGR for providing bias voltages Vb1–Vb5.

**Figure 16:**
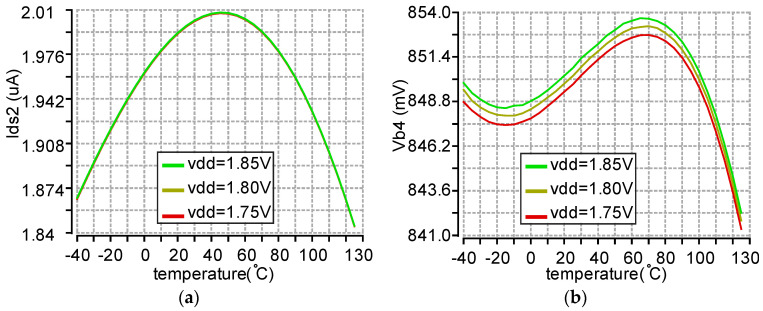
The post layout simulation results of BGR with a temperature range of −40 to 125 °C and vdd range of 1.75–1.85 V. (**a**) The current flow of M2 (1.925 uA ± 81 nA). (**b**) Vb4 (0.85 V ± 3.6 mV).

The bias voltages Vb1–Vb5 used in AFE and ADC are from a simple BGR shown in Figure 15. M8–M10 consist of a startup circuit. Assuming this BGR does not start when the power is on, Va is 0 and Vb is vdd (1.8 V), which will turn on M10 and then M3,4. Finally, Va increases to 1.454 V and turns off M9. Then, Vb decreases to 0 V and turns off M10. The bias voltage, such as Vb4, can be expressed as,

(6) $$V_{i}=(V_{b5}-V_{b4})\frac{C_{1}}{C_{2}}=50mV\cdot \frac{2}{55}=1.82mV$$

where Vth1/(R1 + R2) is the current that flows through M3, 4, or 5. Vth1 is the threshold voltage of M1, which is negative temperature-dependent as is R2 (High-Ohmic N + Poly1). R1 (N-well) is positive temperature-dependent. R1 and R2 can be selected such that the combination of R1 and R2 has the same negative temperature-dependent ratio as Vth1, which makes the current (Vth1/(R1 + R2)) independent of temperature variation, as shown in Figure 16a. This current is mirrored to R3 and R4 from M5. By choosing R3 and R4 properly, the bias voltage Vb4 can be curvature-compensated as shown in Figure 16b. The analysis is the same for Vb3 and Vb5. The bias voltage Vb1 (1.7 V) nears vdd, which needs a low drop output regulator circuit (M7, R9-R12 and one op-amp). A unit gain buffer (not shown here) follows each bias voltage (Vb3–Vb5) to prevent the loading of the bias voltages.

### 2.6. Power-on-Reset Circuit and 500kHz RC Oscillator

**Figure 17:**
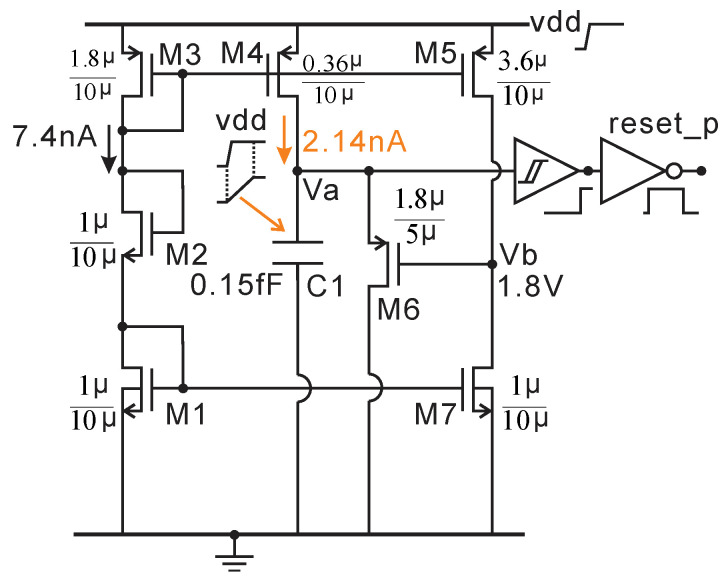
Power-on-reset circuit for AFE and ADC.

**Figure 18:**
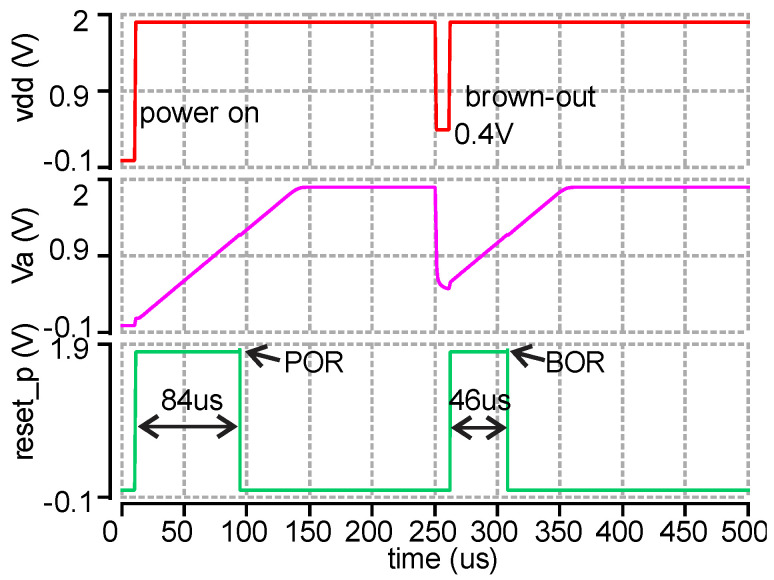
Post layout transient simulation results of the power-on-reset circuit.

The power-on-reset (POR) circuit shown in Figure 17 is used to ensure that digital circuitry and clock signals are initialized correctly on startup. A very small current (2.14 nA) is generated by M1–M4, which produces an 84 µs power-on delay signal (Va) by charging the capacitor C1. The slowly increasing voltage, Va, can be seen in Figure 18-(Va) when the power is on. Then, Va is transferred to a POR signal (84 us) by the following Schmitt trigger and inverter. In addition, M5–M7 are used to produce a brown-out (BOR) signal. If the power supply dips during operation, the BOR can automatically reset the AFE and ADC similar to the POR. The transient simulation results are presented in Figure 18. The 46 us BOR signal is generated after the power drops to 0.4 V. The average current consumption of the POR circuit is 15 nA.

**Figure 19:**
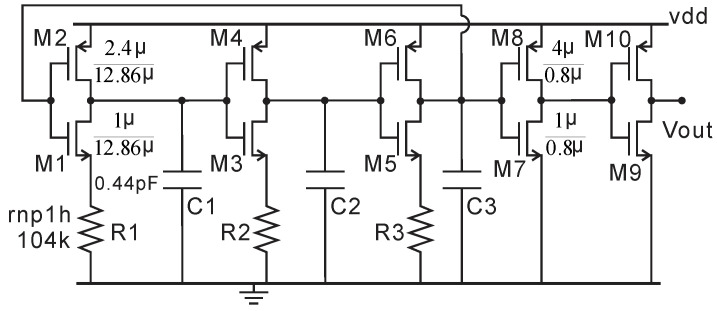
500kHz RC oscillator.

**Figure 20:**
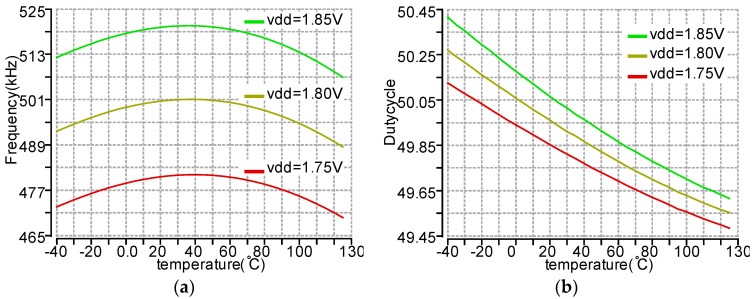
The simulation results of the RC oscillator with a temperature range of −40 to 125 °C and vdd range of 1.75–1.85 V. (**a**) Frequency output. (**b**) Duty cycle.

All the clock signals come from the 500 kHz RC oscillator shown in Figure 19. The threshold voltage of M1, M3, and M5 has a negative temperature-dependent property, as does R1–R3 (High-Ohmic N + Poly1). By choosing the resistance of R1–R3 properly, the temperature variation can be canceled. The duty cycle of the clock is tuned to 50% by sizing the transistors M1–M6. The frequency output and duty cycle versus temperature variation at different power supply are shown in Figure 20. Typically, the vdd is from 1.8 V LDO and very stable. Thus, the frequency (495 ± 6.3 kHz) is sufficiently accurate for our application across temperature at a 1.8 V supply. The duty cycle (50 ± 0.46%) is independent of both power supply and temperature variation. It is worth noting that this RC oscillator features low power consumption, an average of 3.2 uA at 1.8 V supply.

## 3. Performance of this ASIC

**Figure 21:**
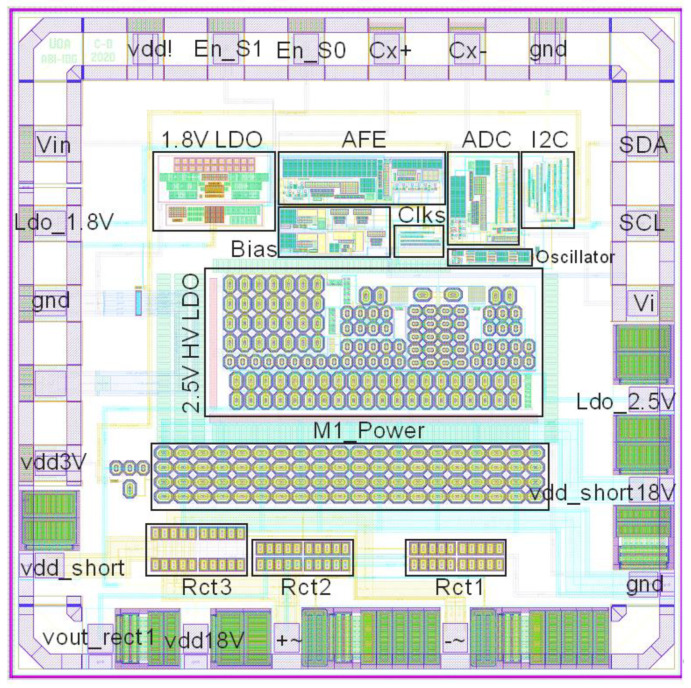
The ASIC (application-specific integrated circuit) layout in 180 nm technology (xh018). The total chip size is 1.5 × 1.5 mm^2^.

**Figure 22:**
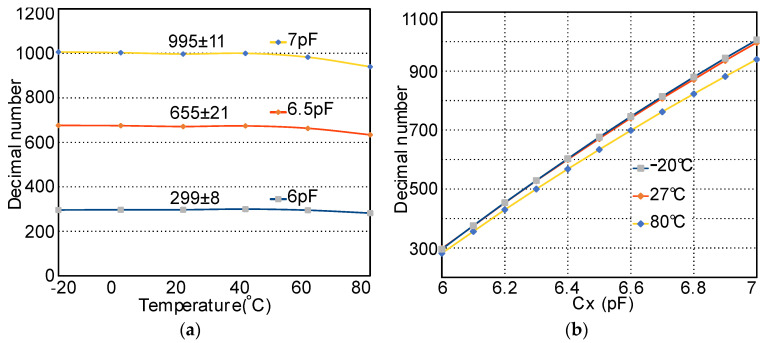
Post layout simulation of C-D digital output. (**a**) Digital output versus temperature with Cx = 6, 6.5, and 7 pF. (**b**) Digital output versus capacitance change with temperature of −20, 27, and 80 °C.

The layout of this ASIC is shown in Figure 21, which has been sent for fabrication. The pads Vin and Vi are the AFE output and integrator’s output, respectively. “+~” and “−~” are two special ESD pads for AC input and can work at a voltage range of −18 to 42 V. The post layout simulation of AFE, ADC, and I2C with different capacitance and temperature is presented in Figure 22. When Cx is unchanged, the digital output is only modestly affected by the temperature, as shown in Figure 22a. The worst-case accuracy owing to the temperature variation (−20 to 80 °C) is approximately ±3.2% occurring with an input capacitance of Cx = 6.5pF, which improves to ±0.86% when operated between 20 and 60 °C. With smaller temperature variation, this accuracy could be better. The digital number steadily increases from 299 to 995 as Cx rises from 6 to 7 pF (300–1000 mmHg), as depicted in Figure 22b, which equals 1.4 fF (0.98 mmHg) resolution or 9.4 Effective Number Of Bits (ENOB).

The performance comparison of the proposed wirelessly powered C-D is presented in Table 1. [7] and [12] have a better resolution than the current work but require higher power consumption and have lower data rates. Although [4,13] have lower power consumption, they do not achieve the same resolution or pressure range. Temperature dependence cannot be compared, as it was not presented in [4,7,12,13]. Compared with previous academic work with the same function, this C-D has the advantage of low-temperature sensitivity, wide pressure range, and a comparable trade-off between resolution and power consumption. More detail about the power consumption on each block is described in Table 2.

## 4. Conclusions

The complete process of designing a low-power capacitive pressure sensor interface IC with wireless power and data transfer in a standard 0.18 µm CMOS technology has been described in the system and transistor levels. This IC is powered by an inductive power transfer circuit. The capacitance change of the sensor is converted into a digital number by the AFE and single slope ADC. Wireless data transfer is achieved through inductive backscatter with a 500 kHz modulated digital signal from the ADC. This AFE, ADC, bias, and oscillator fulfill a 0.98 mmHg resolution and consume an average current of 180 µA at 1.8 V supply.

To optimize the resolution, a delta-sigma ADC can be designed to replace the single-slope ADC in future work. In addition, we will apply this IC to minimally invasive implantable pressure sensors to monitor pulmonary artery pressure.

## Figures and Tables

**Table 1:** Performance comparison of the interface circuits.

Pressure Sensor Interface	This Work	JSSC [4] 2011	TBE [7] 2010	JSSC [12] 2018	JSSC [13] 2009
Technology (µm)	**0.18**	0.13	0.13	0.18	1.5
Supply (V)	**1.8**	1.5	2.2	2.1	2
Temperature (°C)	**−20 to 80**	27–45	NA	NA	NA
Capacitance	**6~7**	6.4–6.5	5.23–5.56	10–12 pF	2–2.2
(pF)					
Pressure range (mmHg)	**300~1000**	750–817	760–810	600–1100	750–950
Resolution	**0.98**	1.32	0.5	0.78	1
(mmHg)					
AFE	**SC**	C-F	C to time	SC	SC
ADC	**Single slope**	No need	Schmitt trigger	SAR	Cyclic
Wireless power	**Inductive**	Inductive RF	mRF	Ultrasonic	Inductive RF
Wireless data transfer	**backscatter**	backscatter	mRF	Ultrasonic	FSK
Data rate (kHz)	**0.05**	NA	0.2	0~1	1
Power (W)	**7.8 m**	2.3 µ	3.2	800 m	36 µ

**Table 2:** Power consumption of each block.

Pressure Sensor Interface	Bias Circuit	AFE	ADC	Oscillator	Power on Reset	1.8 V LDO	2.5 V HV LDO	Data Transmission	Total
Voltage (V)	1.8	1.8	1.8	1.8	1.8	2.5	4.2	2.38 (rms)	**-**
Current (µA)	52	68	56.8	3.2	0.015	42.5	94	2.95 m (rms)	**3.27 m**
Power (µW)	93.6	122.4	102.24	5.76	0.027	106.25	394.8	7 m	**7.8 mW**
